# Senecavirus A- and Non-Infected Cells at Early Stage of Infection: Comparative Metabolomic Profiles

**DOI:** 10.3389/fcimb.2021.736506

**Published:** 2022-01-07

**Authors:** Fuxiao Liu, Bo Ni, Rong Wei

**Affiliations:** ^1^ College of Veterinary Medicine, Qingdao Agricultural University, Qingdao, China; ^2^ Surveillance Laboratory of Livestock Diseases, China Animal Health and Epidemiology Center, Qingdao, China

**Keywords:** metabolomics, senecavirus A, metabolite, metabolic pathway, pyrimidine metabolism, KEGG enrichment analysis, TCA cycle

## Abstract

Senecavirus A (SVA), classified into the genus *Senecavirus* in the family *Picornaviridae*, causes an infectious disease in pigs. This virus can efficiently replicate in some non-pig-derived cells, such as the BHK cell line and its derivative (BSR-T7/5 cell line). We had recovered a wild-type SVA from its cDNA clone previously, and then uncovered the proteomic profile of SVA-infected BSR-T7/5 cells at 12 h post inoculation (hpi). In order to explore the cellular metabolomics further, the SVA-inoculated BSR-T7/5 cell monolayer was collected at 12 hpi for assay *via* liquid chromatography-tandem mass spectrometry (LC-MS/MS). The resultant data set was comprehensively analyzed using bioinformatics tools. A total of 451 metabolites were identified using in-house and public databases. Out of these metabolites, sixty-one showed significantly differential values (*p* value < 0.05). The Kyoto Encyclopedia of Genes and Genomes (KEGG) database was used to analyze metabolic pathways of the significantly differential metabolites. There were eighty-one identified KEGG pathways, out of which twenty-seven showed their *p* values < 0.05. The pyrimidine metabolism revealed the minimum *p* value and the maximum number of significantly differential metabolites, implying the pyrimidine played a key role in cellular metabolism after SVA infection. SVA replication must rely on the cellular metabolism. The present study on metabolomics would shed light on impacts of SVA-induced multiple interactions among metabolites on cells or even on natural hosts.

## 1 Introduction

Senecavirus A (SVA), formerly known as Seneca Valley virus, is an emerging virus that causes vesicular disease and epidemic transient neonatal losses in pigs. This virus belongs to the genus *Senecavirus* in the family *Picornaviridae*. It is the only member in the genus *Senecavirus*. Mature virion is a non-enveloped icosahedral particle with a diameter of approximately 27 nm. The viral genome is a positive-sense, single-stranded and nonsegmented RNA, approximately 7300 nucleotides (nt) in length, with a 3’ poly (A) tail but without a 5’ capped structure. The genome contains 5’ and 3’ untranslated regions, and a single long open reading frame (ORF) of polyprotein precursor. The polyprotein precursor can be cleaved stepwisely into twelve polypeptides, namely the L, VP4, VP2, VP3, VP1, 2A, 2B, 2C, 3A, 3B, 3C^pro^ and 3D^pol^ (Liu et al., 2019; Liu et al., 2020b).

The baby hamster kidney (BHK) and porcine kidney (PK-15) cell lines are generally used for isolation and cultivation of SVA. The BSR-T7/5 was derived from the BHK cell line. We found that the cell-adapted SVA could induce a robust cytopathic effect (CPE) on a BSR-T7/5 cell monolayer at 24 h post inoculation (hpi) or even earlier (Liu et al., 2021a). SVA is also an oncolytic virus with selective tropism for some human tumors with neuroendocrine characteristics (Reddy et al., 2007; Morton et al., 2010; Poirier et al., 2013; Liu et al., 2021c). Anthrax toxin receptor 1 (ANTXR1) was initially discovered as a tumor endothelial marker, also known as tumor endothelial marker 8 (TEM8) (Carson-Walter et al., 2001). The ANTXR1 has been recently recognized as a cellular receptor for SVA infection (Miles et al., 2017; Cao et al., 2018). SVA virion can interact specifically with the ANTXR1 for initiating endocytosis to invade a permissive cell, and then the viral genome would be released from an early endosome to bind to the ribosome for encoding protein.

We demonstrated that enhanced green fluorescent protein (eGFP)-tagged SVA could initiate eGFP expression identifiable as early as 6 hpi in cells. Fluorescence became increasingly bright from 6 to 9 hpi, and was relatively stable after 9 hpi (Liu et al., 2020a). SVA generally induces unobservable CPE on cell monolayers within 12 hpi, during which the virus-infected cells, however, underwent significant variation in several metabolic pathways, like carbon, purine and pyrimidine metabolisms, as evidenced by our recent report concerning proteomic profiling for SVA-infected cells (Liu et al., 2021b). SVA infection triggers a variety of metabolic and biochemical changes in its host cells through virus-specific or -nonspecific mechanisms (Fernandes et al., 2019; Hou et al., 2019; Wen et al., 2019; Li et al., 2020). Integrated omics can assist on deciphering complex networks *in vitro* or *in vivo*, and especially on unveiling the interaction among bio-relevant molecules to affect disease outcome (Rosa-Fernandes et al., 2019). Besides our recent proteomics study on SVA-infected cells (Liu et al., 2021b), Wang et al. (2020) had reported earlier a transcriptomic profile of SVA-infected pig kidney cells (Wang et al., 2020).

Virions are non-living entities, as such intrinsically have no their own metabolism, and nevertheless, are able to modify dramatically the cellular metabolism after they invade permissive cells. It is necessary for identifying how virus infection alters the cellular metabolism or where the metabolic change occurs in a virus-infected host. Uncovering virus-induced changes in the cellular metabolism will not only provide a deeper understanding of viral replication needs, but also reveal potential targets for designing drugs to inhibit viruses (Sanchez and Lagunoff, 2015). In general, researchers focus not only on the variation in a few metabolites, but also on a comprehensive profile of all metabolites in virus-infected cells. In other words, metabolomics, the comprehensive and quantitative analysis of all metabolites in a biological system (Fiehn, 2002), is much more meaningful than single metabolites for the virological research.

Since its initial introduction in 1999 (Nicholson et al., 1999), metabolomics has been found to be applicable to a wide range of fields, including the study of gene function, toxicology, microbiology, clinical diagnostics, and the discrimination of organism genotypes (Jones and Cheung, 2007). In the virological field, the first metabolomics study was reported in 2006, and analyzed over sixty metabolites during human cytomegalovirus infection (Munger et al., 2006). Since then, a variety of viruses have been demonstrated to alter multiple metabolic pathways in cells, like glycolysis and fatty acid synthesis, and have expanded the number of cellular metabolites (Birungi et al., 2010; Ritter et al., 2010; Hollenbaugh et al., 2011; Delgado et al., 2012; Sanchez et al., 2017; Tian et al., 2019; Li et al., 2021; Nunes et al., 2021; Pant et al., 2021; Wang et al., 2021). We have constructed a wild-type SVA (CH-LX-01-2016) using reverse genetics (Liu et al., 2021a), and more recently have revealed a proteomic profile of BSR-T7/5 cells inoculated with this virus at 12 hpi (Liu et al., 2021b). In the present study, in order to uncover its metabolomic profile, the SVA-inoculated BSR-T7/5 cell monolayer was collected at 12 hpi for assay *via* liquid chromatography-tandem mass spectrometry (LC-MS/MS). The resultant data set was deeply analyzed using different bioinformatics tools, elucidating major variations in cellular metabolites at the early stage of SVA infection.

## 2 Materials and Methods

### 2.1 Cell Line and Virus

The BSR-T7/5 cells (Buchholz et al., 1999) were cultured at 37°C with 5% CO_2_ in Dulbecco’s modified Eagle’s medium (DMEM), supplemented with 4% fetal bovine serum and containing penicillin (100 U/mL), streptomycin (100 µg/mL), amphotericin B (0.25 µg/mL) and G418 (500 µg/mL). The wild-type SVA was rescued previously from a cDNA clone (Liu et al., 2021a), genetically derived from an isolate, CH-LX-01-2016 (Genbank access No.: KX751945) (Zhao et al., 2017).

### 2.2 Sample Preparation

BSR-T7/5 cells were seeded into twelve T75 flasks for culture at 37°C. Six cell monolayers at 90% confluency were separately inoculated with the passage-5 SVA (multiplicity of infection = 2.5), and the other six served as non-infected controls. There were six SVA-infected samples (S1 to 6) and six non-infected controls (C1 to 6). At 12 hpi, SVA- and non-infected supernatants were removed from flasks. Cell monolayers were gently washed with D-PBS three times, and then independently harvested into sterile centrifuge tubes using cell scrapers. Cell pellets were collected by centrifugation at 2000 rpm for 5 min at 4°C.

### 2.3 Metabolite Extraction

Cell pellets were lysed with 1 ml of ice-cold extraction buffer (methanol: acetonitrile: water 2: 2: 1 v/v), vortexed for 30 s at 4°C, and supersonicated in an ice-water bath for 1 h. The lysates were subsequently incubated at −20°C for 1 h, followed by centrifugation at 16,000 g for 20 min at 4°C for harvesting supernatants, dried in a vacuum centrifugal concentrator. The dried products were separately resuspended in 100 μL of buffer (acetonitrile: water 1: 1 v/v), followed by centrifugation at 16,000 g for 20 min at 4°C for LC-MS/MS analysis.

### 2.4 Liquid Chromatography Separation

Samples were separated on an ultra-high-pressure liquid chromatography (UHPLC) system (Agilent 1290 Infinity LC, Agilent Tech., CA, the USA) with an ACQUITY UPLC BEH Amide column (2.1 mm × 100 mm, 1.7 μm, Waters), at a column temperature of 25°C, flow rate of 0.3 mL/min and injection volume of 5 μL. Samples were placed in a 4°C autosampler throughout the process. The mobile phase A consisted of a mixture of 25 mM ammonium acetate and 25 mM ammonia hydroxide in water (pH = 9.75), and the mobile phase B was acetonitrile. The analysis was carried out with an elution gradient as follows: 0~0.5 min, 95% B; 0.5~7.0 min, B decreasing linearly from 95 to 65%; 7.0~9.0 min, B decreasing linearly from 65 to 40%; 9.0~10.0 min, B being maintained at 40%; 10.0~11.1 min, B increasing linearly from 40 to 95%; 11.1~16.0 min, B being maintained at 95%. In order to monitor and to evaluate the stability of system and the reliability of experimental data, quality control (QC) samples were inserted into the sample queue.

### 2.5 Mass Spectrometry Detection

The samples were separated by UHPLC, and then subjected to mass spectrometry using the Triple TOF 5600 mass spectrometer (AB Sciex, Toronto, Canada). Mass spectrometry detection was carried out as described previously (Tian et al., 2019) with slight modifications. Briefly, the electrospray ionization (ESI) positive and negative ion modes were used for mass spectrometry detection. The ESI source conditions were as follows: Ion Source Gas1 (Gas1): 60 psi, Ion Source Gas2 (Gas2): 60 psi, Curtain gas (CUR): 30 psi, source temperature: 600°C, IonSapary Voltage Floating (ISVF) ± 5500 V; TOF MS scan m/z (mass-to-charge ratio) range: 60 to 1200 Da, product ion scan m/z range: 25 to 1200 Da, TOF MS scan accumulation time: 0.15 s/spectra, and product ion scan accumulation time: 0.03 s/spectra; the information-dependent acquisition (IDA) mode of the mass spectrometer was used to acquire MS/MS spectra with high sensitivity mode; declustering potential (DP): ± 60 V; collision energy range: 30 eV; the IDA parameters were set as follows, exclude isotopes with 4 Da, and candidate ions to monitor per cycle: 6.

### 2.6 Data Processing and Statistical Analysis

Data processing was performed as described previously (Tian et al., 2019) with slight modifications. In brief, raw data were analyzed by the R package XCMS for peak alignment, calibration, and retention time peak area extraction. Metabolite structure identification used a method of accurate mass matching (<25 ppm). Multivariate analyses were carried as described previously (Mavel et al., 2013; Diémé et al., 2015). The SIMCA-P 14.1 (Umetrics, Umea, Sweden) was used to establish a statistical model. The data were preprocessed by unit variance (UV) scaling for multidimensional statistical analysis, including unsupervised principal component analysis (PCA), supervised partial least squares discriminant analysis (PLS-DA), and orthogonal partial least squares discriminant analysis (OPLS-DA). The quality of model was described by the cumulative modeled variation in the X matrix R^2^X, the cumulative modeled variation in the Y matrix R^2^Y, and the cross-validated predictive ability Q^2^ values (Mavel et al., 2013; Nadal-Desbarats et al., 2014). Single-dimensional statistical analysis included Student′s *t*-test and variation multiple analyses. PCA maps, volcano maps, and cluster maps were generated with the R program.

### 2.7 Differential Metabolite Analysis and Functional Pathway Analysis

Differential metabolite analysis and functional pathway analysis were also carried out as described previously (Tian et al., 2019). The characteristics of metabolite expression patterns were used to explore the differential metabolites with biological significance, through the variable importance for the projection (VIP). VIP > 1 was selected as the screening standard, and the differences between the groups were initially screened. Univariate statistical analysis was used to confirm significant differences in the levels of metabolites. Differential metabolites were identified by adjustments of the *p* value for multiple testing at both VIP > 1 and univariate statistical analysis *p* value < 0.05.

The differential metabolites were analyzed using the MetaboAnalyst 5.0 (https://www.metaboanalyst.ca) (Pang et al., 2021). Data were uploaded to the Kyoto Encyclopedia of Genes and Genomes (KEGG) web service (https://www.kegg.jp) and the human metabolome database (HMDB) 4.0 (https://hmdb.ca) (Wishart et al., 2018) for obtaining more information to identify significantly altered pathways. The SMPDB (https://smpdb.ca/) was used for elucidating metabolic pathways by the metabolite set enrichment analysis (MSEA) based on over representation analysis (ORA). All these programs support a variety of complex statistical calculations and high-quality graphic rendering capabilities that require copious computing resources.

## 3 Results

### 3.1 QC and Quality Assurance (QA) of LC–MS/MS

**Figure 1:**
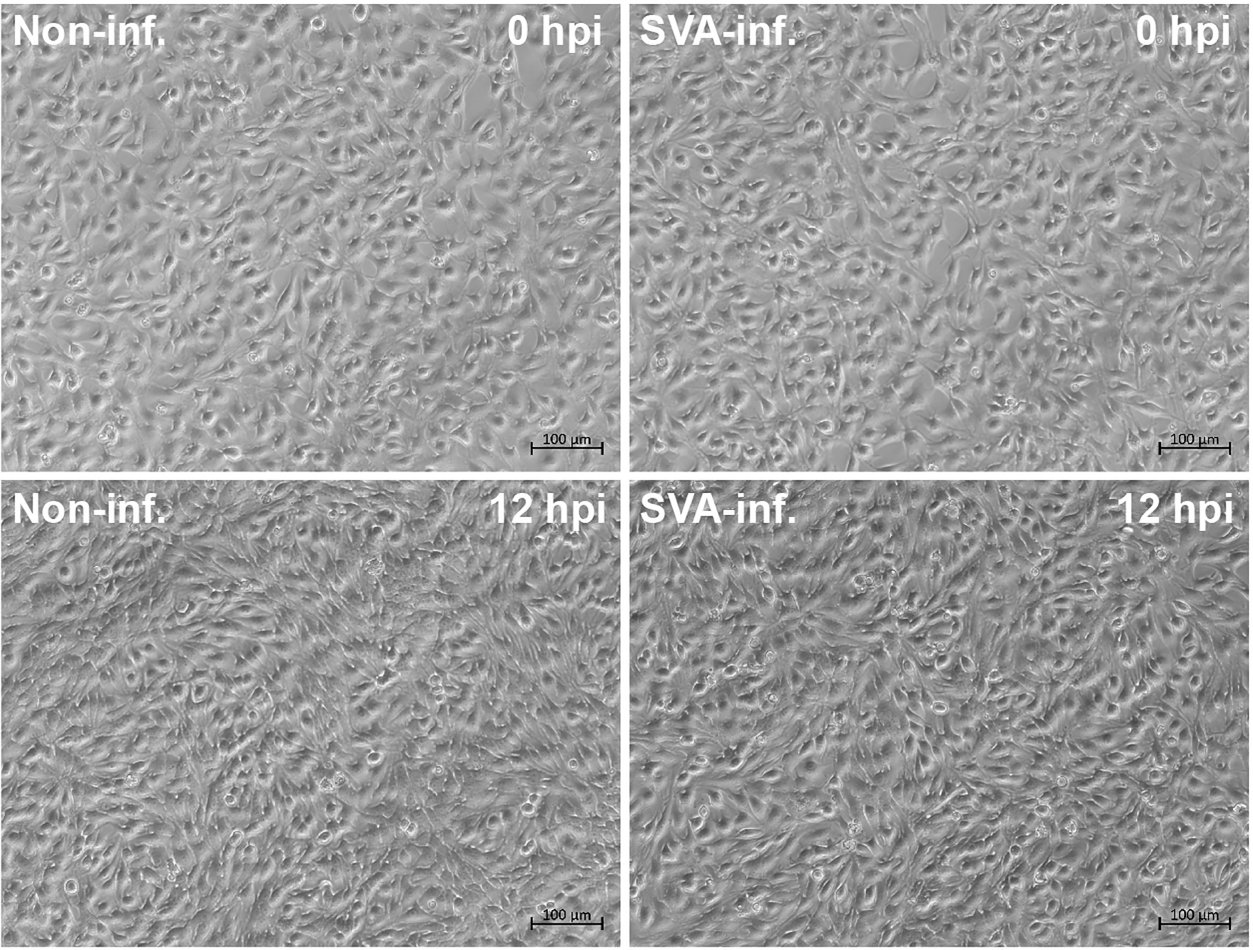
SVA- and non-infected BSR-T7/5 cell monolayers at 0 and 12 hpi. The rescued wild-type SVA at passage-5 is used for inoculation of cells at multiplicity of infection of 2.5.

**Figure 2:**
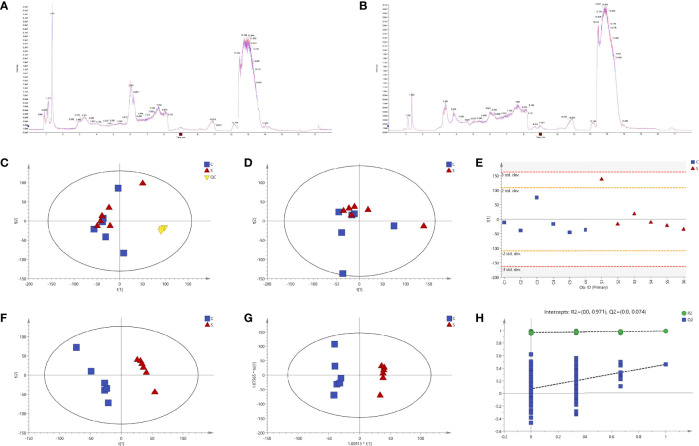
Quality control (QC) and multivariate statistical analyses. Total ion chromatograms (TICs) of QC samples in positive **(A)** and negative **(B)** ion modes. Model of unsupervised principal component analysis (PCA) with **(C)** or without **(D)** QC samples. Score plot of the first principal component (PC1) **(E)**. Supervised partial least squares discriminant analysis (PLS-DA) **(F)**. Orthogonal partial least squares discriminant analysis (OPLS-DA) **(G)**. A 200-time permutation test for evaluating the OPLS-DA model **(H)**.

SVA induced no typical CPE on BSR-T7/5 cells at 12 hpi (**Figure 1**). Cells were separately collected from the six samples (S1 to 6) and six controls (C1 to 6) for metabolomic profiling. Total ion chromatograms (TICs) of QC samples were compared with one another in positive (**Figure 2A**) and negative (**Figure 2B**) ion modes, showing highly overlapping response intensity and peak retention time. The correlation coefficients of QC samples were more than 0.9, suggesting a good correlation with highly reliable data, and also implying that the analysis system was highly stable and repeatable. The MS-DIAL software was used for extracting the pure MS/MS spectrum of metabolites, including 7402 positive- and 5794 negative-ion peaks, followed by UV scaling for PCA. The 7-fold cross-validation was used to construct the PCA model, on which three QC samples were nearly gathered together at the same position (**Figure 2C**), indicating relatively high repeatability in this test.

### 3.2 Multivariate Statistical Analyses

Multivariate statistical analyses, including PCA, PLS-DA and OPLS-DA, were performed to test the reliability of the obtained sample data. The PCA score plot (**Figure 2D**) did not reveal tightly clustered S and C groups [R^2^X (cumulative) = 0.399]. **Figure 2E** was the score plot of PC1. PLS-DA and OPLS-DA were two supervised statistical methods of discriminant analysis. Both the PLS-DA (**Figure 2F**) and the OPLS-DA (**Figure 2G**) showed that the S and C groups were clearly separated and had no overlap, suggesting that both possessed different metabolomic profiles. A 200-time permutation test was conducted to evaluate the OPLS-DA model (**Figure 2H**).

### 3.3 Differential Metabolite Analysis

**Figure 3:**
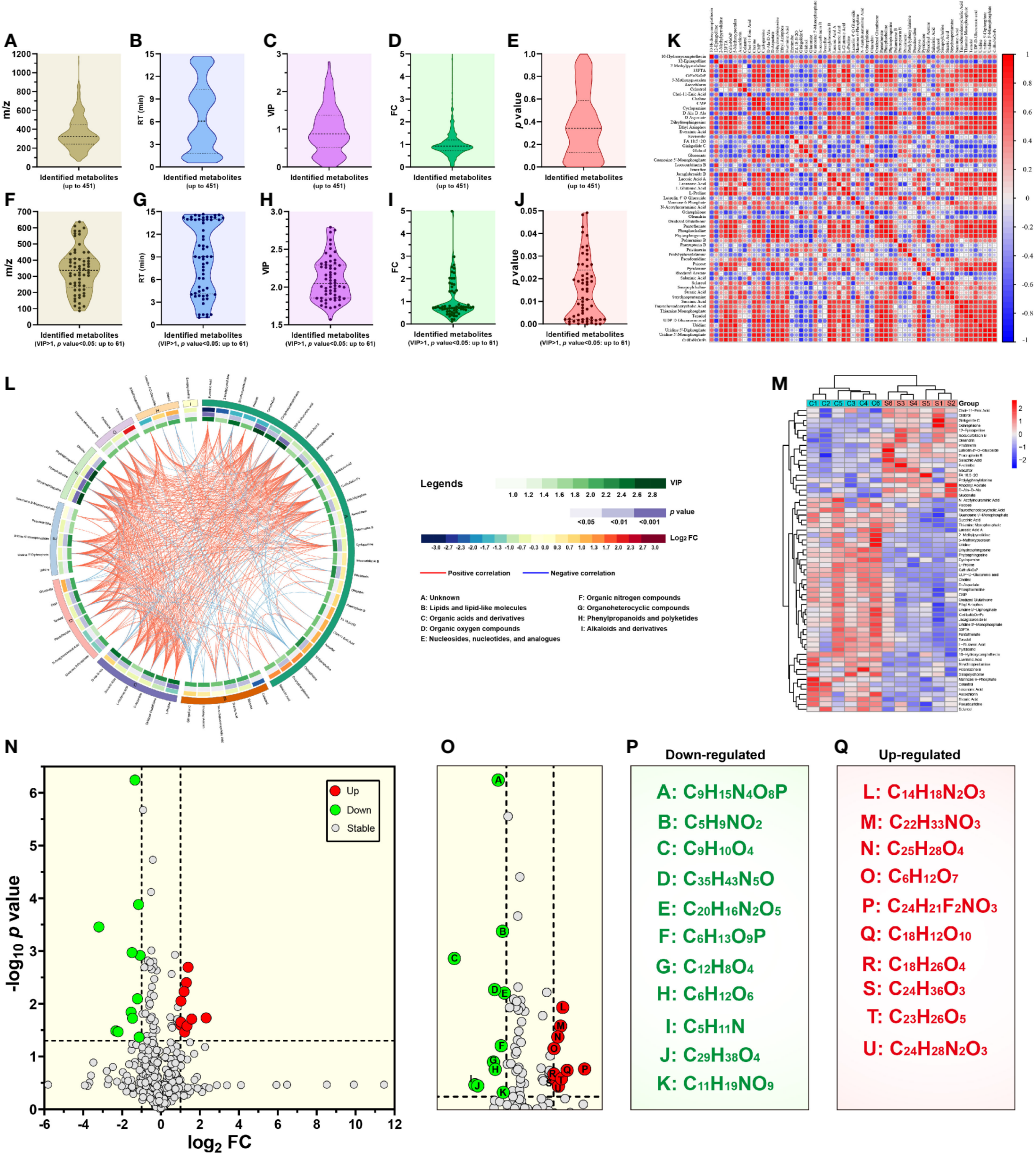
Metabolomic profiling and differential metabolite analysis. Data distributions of mass-to-charge ratio (m/z) **(A)**, retention time (RT) **(B)**, variable importance for the projection (VIP) **(C)**, fold change (FC) **(D)** and *p* value **(E)** for all 451 identified metabolites. Data distributions of m/z **(F)**, RT **(G)**, VIP **(H)**, FC **(I)** and *p* value **(J)** for 61 significantly differential metabolites. Matrix heatmap based on calculation of Pearson’s correlation coefficients for significantly differential metabolites **(K)**. −1 < Pearson’s correlation coefficient (R) < 1. Chord diagram based on calculation of Pearson’s correlation coefficients for significantly differential metabolites **(L)**. 0.7 < absolute value of Pearson’s correlation coefficient (R). Heatmap showing hierarchical clustering of significantly differential metabolites **(M)**. Volcano plot revealing *p* value *versus* FC for all 451 identified metabolites **(N)**. The threshold values are set as VIP > 1.0, FC > 2.0 or FC < 0.5, and *p* value < 0.05. Locally enlarged volcano plot that highlights eleven downregulated (letter A to K in green circles) and ten upregulated (letter L to U in red circles) metabolites **(O)**. Formulas of eleven downregulated metabolites **(P)**. Eleven generic names of metabolites, corresponding to their formulas, are listed in **Supplementary File 1**. There are two isomers: C_6_H_12_O_6_ (psicose) and C_5_H_11_N (2-methylpyrrolidine). Formulas of ten upregulated metabolites **(Q)**. Ten generic names of metabolites, corresponding to their formulas, are also listed in **Supplementary File 1**.

Metabolites between different groups were screened using in-house and public databases, the latter including the HMDB (https://hmdb.ca/) and the MassBank (http://www.massbank.jp). A total of 451 metabolites were identified, and listed in **Supplementary File 1**. **Figures 3A–E** exhibited data distributions of m/z, retention time (RT, min), VIP, fold change (FC) and *p* value for all 451 metabolites, respectively. Out of 451 identified metabolites, sixty-one showed significantly differential values (**Table 1**), namely VIP > 1 and *p* value < 0.05. **Figures 3F–J** exhibited data distributions of m/z, RT (min), VIP, FC and *p* value for these sixty-one metabolites, respectively. To uncover functions of the characteristic metabolites at the early stage of SVA infection, differential analyses were performed to analyze the metabolite profiles in SVA- and non-infected cells.

**Table 1:** Metabolites with significantly different values (VIP > 1 and *p* value < 0.05) between SVA- and non-infected BSR-T7/5 cells.

No.	ESI mode	RT (min)	m/z	Formula*	VIP	FC (S/C)	*p* value
1	Negative	13.485	362.98	C_20_H_16_N_2_O_5_	2.41196	0.473	0.001208394
2	Positive	3.462	360.25	C_22_H_33_NO_3_	2.2432	2.463	0.003971111
3	Positive	10.489	86.10	C_5_H_11_N	1.75889	0.199	0.032614933
4	Negative	11.719	241.01	C_6_H_5_F_7_O_2_	2.04971	0.655	0.010868178
5	Negative	14.196	337.05	C_9_H_15_N_4_O_8_P	2.79392	0.395	5.72E-07
6	Negative	8.937	215.03	C_12_H_8_O_4_	1.98074	0.343	0.014427207
7	Negative	3.812	403.27	C_23_H_29_ClO_4_	2.08883	0.724	0.017452802
8	Negative	3.763	449.27	C_29_H_38_O_4_	1.82637	0.217	0.03404002
9	Negative	10.064	371.26	C_24_H_36_O_3_	1.85575	2.024	0.025508359
10	Positive	8.046	104.11	C5H_14_NO	2.24857	0.858	0.002328677
11	Negative	14.297	322.04	C_9_H_14_N_3_O_8_P	2.28817	0.764	0.001579191
12	Positive	6.139	412.32	C_27_H_41_NO_2_	2.29465	0.769	0.002006941
13	Positive	4.436	161.09	C6H_12_N_2_O_3_	2.45242	1.642	0.001182334
14	Negative	14.165	132.03	C_4_H_7_NO_4_	2.26052	0.715	0.002229143
15	Positive	7.243	302.30	C_18_H_39_NO_2_	2.62737	0.702	7.60E-05
16	Positive	14.291	346.04	C_12_H_16_N_3_O_3_PS_2_	2.12955	0.723	0.004767953
17	Negative	3.875	181.10	C_9_H_10_O_4_	2.51068	0.109	0.000349695
18	Negative	4.873	408.14	C_24_H_21_F_2_NO_3_	1.94642	4.984	0.018553072
19	Negative	3.383	305.17	C_18_H_26_O_4_	2.01907	2.021	0.022152116
20	Negative	2.818	439.12	C_20_H_24_O_11_	2.00759	1.813	0.011533487
21	Positive	4.187	393.21	C_25_H_28_O_4_	2.16223	2.267	0.005851153
22	Negative	14.206	195.05	C6H_12_O_7_	2.17888	2.029	0.008878564
23	Negative	14.469	362.05	C_10_H_14_N_5_O_8_P	1.7344	0.849	0.03125592
24	Positive	9.036	581.31	C_32_H_46_O_8_	1.83047	1.442	0.026361225
25	Positive	4.014	393.22	C_24_H_28_N_2_O_3_	1.85159	2.310	0.034457447
26	Positive	13.976	637.13	C_30_H_30_O_13_	2.31837	0.623	0.001506311
27	Positive	13.919	560.08	C_26_H_19_NO_12_	1.75543	0.601	0.040581007
28	Positive	13.975	357.05	C_16_H_14_O7	2.30969	0.661	0.001989898
29	Negative	13.916	146.05	C_5_H_9_NO_4_	1.73593	0.739	0.039123254
30	Negative	11.3	114.06	C_5_H_9_NO_2_	2.56985	0.449	0.000131365
31	Negative	5.02	447.13	C_21_H_20_O_11_	1.94695	1.742	0.020969913
32	Negative	14.562	259.02	C_6_H_13_O_9_P	2.10553	0.430	0.008019549
33	Negative	10.335	308.10	C_11_H_19_NO_9_	1.5696	0.458	0.04291444
34	Negative	1.35	381.17	C_23_H_26_O_5_	1.84786	2.522	0.026272365
35	Positive	9.04	599.32	C_32_H_48_O9	1.81614	1.490	0.028439393
36	Negative	14.513	611.14	C_20_H_32_N_6_O_12_S2	2.34391	0.553	0.002039118
37	Negative	9.047	218.10	C_9_H_17_NO_5_	2.08852	0.646	0.008486492
38	Positive	14.535	184.07	C5H_15_NO_4_P	2.35855	0.707	0.000977563
39	Positive	7.655	318.30	C_18_H_39_NO_3_	2.7277	0.745	1.86E-05
40	Positive	1.04	377.33	C_18_H_23_N_3_O_2_S_2_	1.8566	0.763	0.030887859
41	Positive	4.01	449.15	C_24_H_26_O_7_	1.95456	1.778	0.019583191
42	Negative	3.858	463.28	C_30_H_40_O_4_	2.28288	1.455	0.002728805
43	Negative	8.21	261.13	C_14_H_18_N_2_O_3_	1.78612	2.616	0.002029154
44	Negative	8.706	243.06	C_9_H_12_N_2_O_6_	1.72375	0.709	0.049138388
45	Negative	10.467	179.06	C_6_H_12_O6	1.86924	0.362	0.018833488
46	Negative	13.913	168.03	C_8_H_11_NO_3_	1.82616	0.759	0.026374722
47	Positive	1.342	216.20	C_12_H_22_O_2_	2.00746	1.393	0.018015274
48	Negative	12.502	387.03	C_18_H_12_O_10_	1.99645	2.979	0.019466069
49	Negative	1.356	307.26	C_20_H_36_O_2_	2.19384	0.697	0.006567929
50	Positive	0.954	310.16	C_16_H_24_NO_5_	1.83867	0.753	0.048208933
51	Negative	1.356	283.26	C_18_H_36_O_2_	2.16086	0.774	0.007127692
52	Positive	6.723	550.35	C_35_H_43_N_5_O	2.46787	0.355	0.001069387
53	Negative	13.843	117.02	C_4_H_6_O_4_	2.26628	0.786	0.00339563
54	Negative	4.577	498.29	C_26_H_45_NO_6_S	1.90339	0.801	0.021409706
55	Positive	14.653	345.08	C_12_H_18_N_4_O_4_PS	1.98534	0.516	0.015516282
56	Negative	9.042	254.08	C_15_H_13_NO_3_	2.05772	0.678	0.009940434
57	Negative	14.276	579.03	C_15_H_22_N_2_O_18_P2	2.75407	0.527	2.12E-06
58	Negative	6.179	243.06	C_9_H_12_N_2_O_6_	2.00327	0.573	0.015291785
59	Positive	13.973	405.01	C_9_H_14_N_2_O_12_P_2_	1.89566	0.667	0.020093381
60	Negative	14.207	323.03	C_9_H_13_N_2_O_9_P	2.32867	0.680	0.001628859
61	Negative	13.976	565.04	C_15_H_24_N_2_O_17_P_2_	1.91678	0.703	0.018831091

*Metabolite names, corresponding to their formulas, are listed in **Supplementary File 1**.

#### 3.3.1 Correlation Analysis

Correlation analysis was based on calculation of Pearson’s correlation coefficients, and facilitated the measurement of metabolic proximities among sixty-one significantly differential metabolites (VIP > 1 and *p* value < 0.05). The result of correlation analysis was shown in **Figure 3K** [−1 < Pearson’s correlation coefficient (R) < 1]. Red and blue colors indicated positive and negative correlations, respectively. The darker color and the bigger circle indicated the stronger correlation between two metabolites. The data of correlation analysis were listed in **Supplementary File 2**. Furthermore, all sixty-one metabolites were classified into nine categories *via* the super class analysis of HMDB, namely (1) lipids and lipid-like molecules, (2) organic acids and derivatives, (3) organic oxygen compounds, (4) nucleosides, nucleotides, and analogues, (5) organic nitrogen compounds, (6) organoheterocyclic compounds, (7) phenylpropanoids and polyketides, (8) alkaloids and derivatives, and (9) unknown. The results of correlation analysis were additionally shown in the chord diagram (**Figure 3L**) [0.7 < absolute value of Pearson’s correlation coefficient (R)].

#### 3.3.2 Hierarchical Clustering

The heatmap (**Figure 3M**) offered a visual depiction of the trend of metabolic changes, and showed hierarchical clustering of sixty-one significantly differential metabolites (VIP > 1.0 and *p* value < 0.05). The horizontal and vertical coordinates indicated metabolite names and intensity levels, respectively. Red and blue colors represented high- and low-intensity metabolites, respectively. The intensity of color reflected the degree of change in metabolite intensity.

#### 3.3.3 Univariate Statistical Analysis

The volcano plot was drawn by the GraphPad Prism software (Version 8.0) to display the *p* value *versus* the FC for all 451 identified metabolites (**Figure 3N**). The threshold values were set as VIP > 1.0, FC > 2.0 or FC < 0.5, and *p* value < 0.05. A total of twenty-one metabolites, highlighted in **Figure 3O**, revealed significantly differential changes between SVA- and non-infected cells. Out of these twenty-one metabolites, ten (**Figure 3Q**, red circle-shadowed) and eleven (**Figure 3P**, green circle-shadowed) were upregulated and downregulated, respectively. Each letter-marked circle (**Figure 3O**) represented one downregulated or upregulated metabolite, whose formula was shown in **Figures 3P** or **Q**, respectively. Twenty-one metabolite names, corresponding to their formulas, are listed in **Supplementary File 1**.

### 3.4 Metabolic Pathway Analysis

The KEGG database was used to analyze metabolic pathways of the sixty-one significantly differential metabolites. The substance identification (ID) numbers of KEGG were obtained through the ID conversion function of the MBROLE 2.0 (http://csbg.cnb.csic.es/mbrole2). The KEGG IDs of differential metabolites (**Supplementary File 3**) were used for pathway enrichment analysis through the MBROLE pathway analysis function. There were a total of eighty-one identified KEGG pathways (**Supplementary File 4**), involved in twenty-five metabolites.

**Figure 4:**
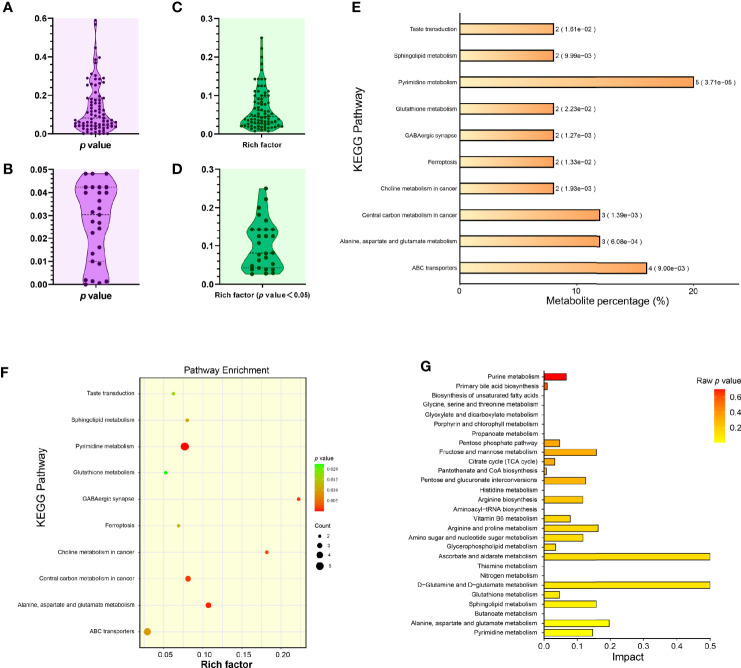
Enrichment analysis of KEGG metabolic pathways related to significantly differential metabolites. Distribution of *p* values corresponding to all identified KEGG pathways **(A)**. Distribution of *p* values corresponding to significantly differential KEGG pathways **(B)**. Distribution of rich factors corresponding to all identified KEGG pathways **(C)**. Distribution of rich factors corresponding to significantly differential KEGG pathways **(D)**. Histogram of top-10 statistically significant KEGG pathways **(E)**. Each bar is followed by the number of significantly differential metabolites with the corresponding *p* value. Metabolite percentage = Count/List Total (**Supplementary File 4**). Bubble plot of top-10 statistically significant KEGG pathways **(F)**. Rich factor = Count/Pop Hit (**Supplementary File 4**). The greater rich factor represents the greater degree of KEGG enrichment. “Impact” comparison among KEGG pathway enrichments **(G)**. The “Impact” means the importance value of metabolic pathways obtained from topological analysis using the MetaboAnalyst 5.0.

**Figure 4A** showed the distribution of *p* values corresponding to these eighty-one pathways, out of which, twenty-seven showed their *p* values < 0.05 (**Figure 4B**). Rich factor was a key parameter, equal to “Count/Pop Hit (**Supplementary File 4**)”. The higher rich factor indicated the greater degree of KEGG enrichment. **Figures 4C, D** showed the distributions of rich factors corresponding to all and significantly differential pathways, respectively. The top-10 significantly differential pathways were shown independently in the histogram (**Figure 4E**) and in the bubble plot (**Figure 4F**). The “pyrimidine metabolism” (Pathway ID: cge00240, and *p* value: 0.0000371) revealed the minimum *p* value (0.0000371), and the maximum number of significantly differential metabolites (up to five).

**Figure 5:**
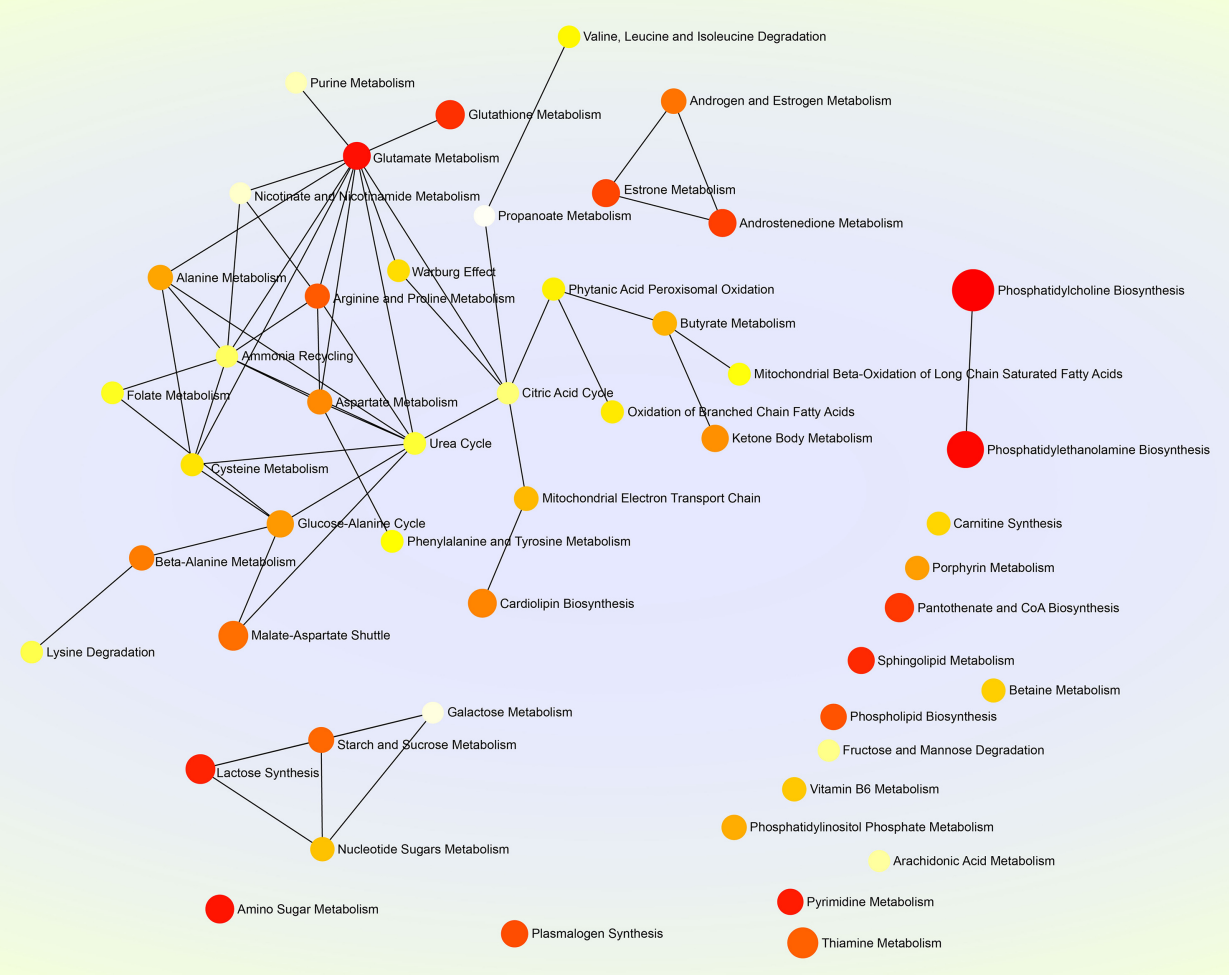
Net map of multiple metabolic pathways. The SMPDB (https://smpdb.ca/) is used for elucidating metabolic pathways by the metabolite set enrichment analysis (MSEA) based on over representation analysis (ORA). Darker and larger nodes represent enriched pathways with lower raw *p* values and with higher enrichment ratios, respectively. Edges represent correlations between metabolic pathways.

MetaboAnalyst 5.0 (https://www.metaboanalyst.ca) was used to perform the pathway enrichment analysis of all sixty-one significantly differential metabolites (**Supplementary File 5**). Subsequently, the package R (version 4.0.3) with the ggplot2 was used to format data, and to plot the histogram (**Figure 4G**), in which the horizontal coordinate represented the importance value of metabolic pathways obtained from topological analysis (**Supplementary File 6**). The SMPDB (https://smpdb.ca/) was used for elucidating the net interaction among metabolic pathways (**Figure 5**) by the ORA-based MSEA (**Supplementary File 7**).

## 4 Discussion

All viruses as such cannot independently replicate to generate their progenies in the nature. Host metabolic machineries, regardless of those *in vivo* or *in vitro*, are required for the viral life cycle, including virion uncoating, genome replication, progeny packaging and so forth. So altered host metabolic pathways can be exploited for containment of these viruses. Metabolomics provides the insight for tracing out such checkpoints (Kumar et al., 2020). Metabolomics is the comprehensive analysis of all metabolites in a single biological system. In the virological field, such a single biological system may be a population of cells, or a natural host. Therefore, so-called “viral metabolomics” is an incorrect concept, and should be replaced with the “cellular metabolomics” or “host metabolomics”. A major aim of metabolomics studies is identification and quantification of small molecules involved in metabolic reactions. Owing to its high throughput, soft ionization, and good coverage of metabolites, the LC-MS/MS technique has enjoyed a growing popularity as the platform for broad metabolomics studies (Zhou et al., 2012).

In the present study, the LC-MS/MS method was used to analyze comparatively metabolomic profiles between SVA- and non-infected BSR-T7/5 cells. The reason why we conducted this study was that our earlier research had preliminarily shown cellular metabolisms, mainly affected in SVA-inoculated cells at the early stage of infection (Liu et al., 2021b). The virus used here was the rescued SVA by means of reverse genetics. We found that this virus could induce robust cell lysis at 24 hpi, resulting in a large number of cellular metabolites were released into culture supernatant. Despite the multiplicity of infection of 2.5, the typical CPE was invisible on the SVA-infected cell monolayer at 12 hpi (**Figure 1**), when SVA actually underwent at least one cycle of replication in cells, as evidenced by SVA virion-like particles packaged in cytoplasm (Liu et al., 2020a). To ensure SVA-infected cells structurally undisrupted, and meanwhile to make virus finish at least one cycle of replication, the time point was set as 12 hpi for harvesting cells. Using the LC–MS/MS technique for metabolomics analysis, we found sixty-one significantly differential metabolites, composed of organic acids, nucleotides, lipids, amino acids, sugars and so on. All metabolites were classified into nine categories, including an unknown one composed of twenty-seven metabolites (**Figure 3L**).

The category “Nucleosides, nucleotides, and analogues” totally contained five members: guanosine 5’-monophosphate (GMP), pseudouridine, uridine 5’-monophosphate (UMP), uridine 5’-diphosphate (UDP), and uridine. Interestingly, four metabolites are uridine and its derivatives, implying that they played a key role in cellular metabolism after SVA infection. Glycosylation in viral proteins or receptors is a highly regulated posttranslational modification, which has multiple effects on viral structure, function, signaling pathways and so on. N-linked glycosylation on anthrax toxin receptor 1 has been recently demonstrated to be essential for SVA infection (Jayawardena et al., 2021). At least four sugar-related metabolites, namely UDP-d-glucuronic acid, uridine-5’-diphosphogalactose disodium salt, luteolin 4’-O-glucoside and oleandrin, were identified here as significantly differential ones between SVA- and non-infected groups in the present study. Their roles in virus-mediated signaling and in regulation of cell growth remain to be elucidated.

In addition to nucleosides and sugars as mentioned above, fatty acids were also important metabolites during viral replication. Many viruses induce and require fatty acid synthesis at some stage of their lifecycle (Sanchez and Lagunoff, 2015). Infection of human cytomegalovirus upregulates much of central carbon metabolic flux, as well as efflux to nucleotide and fatty acid biosynthesis. This unanticipated upregulation of fatty acid biosynthesis is essential for the replication of human cytomegalovirus, influenza A and so forth (Munger et al., 2008). In the present study, the sixty-one significantly differential metabolites include three types of fatty acids, namely medium-chain fatty acid (C_6_H_5_F_7_O_2_), oxidized fatty acid (C_18_H_26_O_4_) and long-chain fatty acid (C_18_H_36_O_2_). The C_18_H_36_O_2_ is stearic acid, a saturated long-chain fatty acid with an 18-carbon backbone. It is involved in metabolism of lipids in cells specially infected with enveloped viruses, like influenza virus (Kordyukova et al., 2008) and measles virus (Anderton et al., 1983a; Anderton et al., 1983b). SVA, albeit classified into one type of enveloped virus, still caused the significantly differential metabolism of stearic acid in infected cells from that in non-infected ones (**Supplementary File 1**).

Glutamate, a key compound in cellular metabolism, plays a vital role in biosynthesis of nucleic acids and proteins. Glutamate metabolism is also closely involved in replication of some viruses in their hosts. For example, glutamate is thought to be concerned with human immunodeficiency virus-induced neurotoxicity (Vázquez-Santiago et al., 2014); replication of white spot syndrome virus depends on glutamate-driven anaplerosis (Li et al., 2016). In the present study, we found the glutamate metabolism correlated with the highest number of metabolic pathways, up to eleven (**Figure 5**), implying its important role in SVA replication *in vitro*, and additionally remaining to be elucidated whether SVA-induced pathologic changes *in vivo* is closely related to it.

The tricarboxylic acid (TCA) cycle is a series of chemical reactions used by aerobic organisms to release stored energy through the oxidation of acetyl-CoA derived from carbohydrates, fats, and proteins. Succinic acid is a dicarboxylic acid, taking the form of an anion, succinate, which has multiple biological roles as a metabolic intermediate being converted into fumarate in the TCA cycle (Tretter et al., 2016). In the present study, the succinic acid belonged to the category “Organic acids and derivatives” (**Figure 3L**). The level of succinic acid was demonstrated to be downregulated after SVA infection, suggesting that the TCA cycle might be affected to some extent. This postulation was confirmed by the following pathway analysis using the MetaboAnalyst 5.0 (**Figure 4G**). Besides the TCA cycle, other five pathways, (1) alanine, aspartate and glutamate metabolism, (2) GABAergic synapse, (3) central carbon metabolism in cancer, (4) butanoate metabolism and (5) carbon metabolism, were significantly affected, and also involved in the succinic acid or succinate. The TCA cycle is closely correlated with several metabolic pathways, *e.g.*, the glutathione metabolism (He et al., 2019; Tompkins et al., 2019). Therefore, the TCA cycle, if affected somewhat in cells, would have a higher or lower impact on other pathways. Indeed, the analysis of KEGG enrichment exhibited that the glutathione metabolism was significantly affected after SVA infection (**Figures 4E, F**). Two significantly downregulated metabolites, L-glutamic acid and oxidized glutathione, were simultaneously enriched into the glutathione metabolism by the KEGG enrichment analysis.

The KEGG enrichment analysis showed a total of twenty-seven metabolic pathways with *p* values < 0.05. Out of them, the pyrimidine metabolism displayed the minimum *p* value (0.0000371), and the maximum number of significantly differential metabolites, *i.e.*, UDP, cytidine 5’-monophosphate (CMP), UMP, uridine and pseudouridine. Host nucleotides and their derivatives are important small molecules, playing a crucial role in cellular metabolism, such as signal transduction, and synthesis of genetic materials (Tian et al., 2019). In our recent proteomics report, the pyrimidine metabolism was also demonstrated to have the lowest *p* value, and however the highest number of significantly differential metabolites were enriched into the purine metabolism (Liu et al., 2021b). Such a slight difference in metabolic profiles between two omics studies might be attributed to distinct methods of experimentation and (or) analysis.

The first case of SVA-infected pigs was found in Canada in 2007. In 2010, a Chester White boar was diagnosed with SVA infection in the USA (Singh et al., 2012). At the end of 2014 and the beginning of 2015, SVA outbreak was repeatedly reported in different regions of Brazil (Leme et al., 2015; Vannucci et al., 2015; Leme et al., 2016a; Leme et al., 2016b). In 2015, this disease was identified in China (Wu et al., 2017), and then gradually spread into other provinces. Now, high- and low-virulence SVAs were simultaneously circulating in China (Liu et al., 2020b). The China isolate, CH-LX-01-2016, had been recovered from its cDNA clone previously (Liu et al., 2021a), and then we deeply explored its role in regulating expression of cellular proteins at the early stage of infection (Liu et al., 2021b). In the present study, we carried out the following metabolomics analysis, and the data set will be expected to be used for unveiling potential metabolite-metabolite interactions that confer special effects, like inducing apoptosis or immune response, on permissive cells or natural hosts.

## Data Availability Statement

The datasets supporting the conclusions of this article are included within the article and its additional files. The metabolomics data have been deposited to the EMBL-EBI MetaboLights database (https://www.ebi.ac.uk/metabolights/index) with the identifier MTBLS3612.

## Author Contributions

Experimental design, RW. Analysis of data, FL and BN. Writing—original draft preparation, FL. Review, BN and RW. Funding acquisition, BN and RW. All authors contributed to the article and approved the submitted version.

## Funding

This work was supported by the Innovation Fund, funded by China Animal Health and Epidemiology Center.

## Conflict of Interest

The authors declare that the research was conducted in the absence of any commercial or financial relationships that could be construed as a potential conflict of interest.

## Publisher’s Note

All claims expressed in this article are solely those of the authors and do not necessarily represent those of their affiliated organizations, or those of the publisher, the editors and the reviewers. Any product that may be evaluated in this article, or claim that may be made by its manufacturer, is not guaranteed or endorsed by the publisher.

## Supplementary Material

Supplementary File S1 Table of ion peaks.

Supplementary File S2 Data of correlation analysis.

Supplementary File S3 KEGG ID mapping.

Supplementary File S4 KEGG Pathway enrichment.

Supplementary File S5 Name map.

Supplementary File S6 Pathway impact.

Supplementary File S7 MSEA ORA result SMPDB.
